# A Selective Dispersion–Suppressed Metasurface for Dual‐Polarization and Dual‐Broadband Electromagnetic Window at Large Angles

**DOI:** 10.1002/nap2.70166

**Published:** 2026-07-20

**Authors:** Tiefu Li, Jinwei Chen, Cunqian Feng, Jian Liu, Yuxiang Jia, Chang Ding, Yajuan Han, Xinmin Fu, Jie Yang, Zhenxu Wang, Song Zhao, Zhaotang Liu, Zhihui Zhang, Jiafu Wang

**Affiliations:** ^1^ Shaanxi Key Laboratory of Artificially‐Structured Functional Materials and Devices Air Force Engineering University Xi'an China; ^2^ National Key Laboratory of Aerospace Flight Technology Air Force Engineering University Xi'an China; ^3^ Air and Missile Defense College Air Force Engineering University Xi'an China; ^4^ Aerospace Metamaterials Laboratory of SuZhou Laboratory Suzhou China

**Keywords:** broadband, dispersion, dual‐polarization, metasurface

## Abstract

The metasurface disperses the surrounding medium with electromagnetic (EM) response, and the desired dispersion endows it with the desired EM function. However, at the same time, the undesired one restricts its working range, especially for transmission metasurfaces. In this manuscript, we propose to suppress the metasurface's undesired dispersion to widen its working range, while preserving the desired one to preserve the function. Through a method of reversed superimposition (RS), we have implemented the idea on our previous work in. It is a metasurface for the dual‐band EM window at large angles (≥ 60°) but only for transverse‐electric (TE) polarization and has a narrow effective band (EB). However, through selective dispersion suppression, the RS metasurface is dual‐polarized and dual‐broadband. This work was well verified through the spectral lines, extracted parameters, and monitored field. Moreover, the RS metasurface was compared with other related works, and the results showed its superiority of dual polarization and broadband. The prototypes were fabricated and measured, where the measured results were basically consistent with the simulated ones. This work may provide new ideas for dual‐polarization and broadband metasurfaces, and find wide applications in 5G/6G base stations, radar systems, and others.

## Graphical abstract

In this manuscript, we propose to suppress the metasurface's undesired dispersion to widen its working range, while preserving the desired one to preserve the function. As shown in the figure, through a method of reversed superimposition (RS), we have implemented the idea on a metasurface for the dual‐band EM window at large angles (≥ 60°). The metasurface is originally only for transverse electric (TE) polarization and has a narrow effective band (EB). However, through selective dispersion suppression, it can work for dual polarization and dual broadband after the RS.

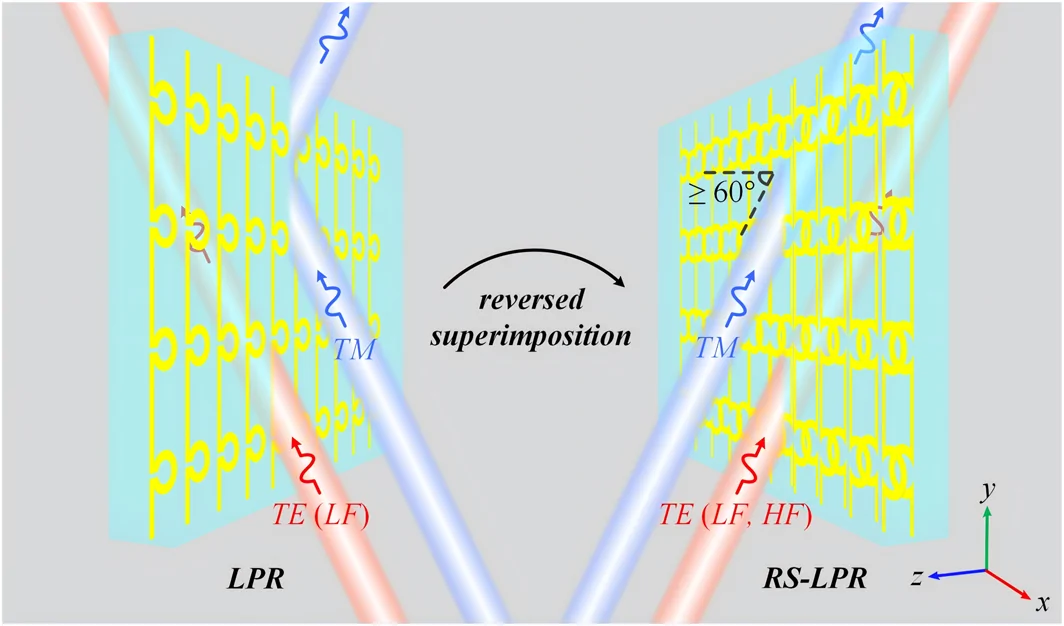

## 1 Introduction

Metamaterials and their special 2D versions, metasurfaces, are artificial materials composed of periodically arranged sub‐wavelength units [1, 2, 3, 4, 5]. With the units' electromagnetic (EM) response, EM waves induce electrical and magnetic field around them to change the field distribution in the surrounding medium. Due to the units' sub‐wavelength dimension, this phenomenon is equivalent to the dispersion of the medium's permittivity and permeability. The desired dispersion endows the metasurface with the desired EM function, such as the negative refractive index [6, 7, 8], wave front manipulation [9, 10, 11, 12, 13, 14, 15], and EM transparency [16, 17, 18, 19, 20, 21, 22, 23, 24, 25, 26, 27, 28]. Therefore, in recent decades, the metasurface has gotten lots of attention in both engineering and academia. However, at the same time, the undesired dispersion restricts its working range. First, for the effective polarization, a metasurface unit generally holds different responses at different (transverse electric (TE) and transverse magnetic (TM)) polarizations, especially at oblique incidence. These responses cause the TE‐ and TM‐polarized dispersions, respectively. Therefore, a metasurface that works for TE polarization may have an undesired TM‐polarized dispersion, thus being ineffective at TM polarization, and vice versa [9, 10, 11, 19, 24, 26, 28, 29]. Second, for the effective band (EB), a response of the unit generally causes multiple dispersions at the same time. However, the metasurface generally works through only one of them, while others are undesired. According to Snell's law, the undesired dispersion would enhance the sensitivity of the metasurface's reflection and transmission to the frequency, thus reducing its EB [9, 10, 11, 12, 13, 14, 15, 24, 28].

With the metal background, the reflection of reflective metasurfaces is nearly 100 % at all frequencies for any polarization, so the above problems are slight [9, 10, 30, 31, 32, 33, 34]. However, for transmission metasurfaces, like Huygens metasurfaces, Brewster metasurfaces, and frequency selective surfaces (FSSs), they would be severe. Compared with reflective metasurfaces, the transmission ones generally work for only one polarization and have a narrower EB [11, 12, 13, 14, 15, 16, 17, 18, 19, 20, 21, 22, 23, 24, 25, 26, 27, 28, 29]. For example, in 2021, Cheng Jin et al. proposed a tunable FSS composed of long metallic strips (LMSs) and patch resonators, which could open an ultra‐wide‐angle EM window in the C band. However, with the resonators' undesired dispersion at TM polarization, it is only for TE polarization [19]. In the same year, Zhe Zhang et al. designed a Brewster metasurface to realize the generalized Brewster effect by the generalized Kerker effect. The design could work for both TE and TM polarization, but its EB is very narrow [21]. Recently, as shown in the left of Figure 1, we have introduced parasitic rings (PRs) into the LMSs to design an LPR metasurface. Through a desired permittivity dispersion, the metasurface could open the dual‐band EM window on the substrate at large incident angles (≥ 60°). However, due to an undesired TM‐polarized dispersion, it is only for TE polarization in the high‐frequency (HF) working band. Additionally, the metasurface has also an undesired TE‐polarized dispersion in the HF band, which has resulted in the narrow EB of the HF window [28].

**FIGURE 1:**
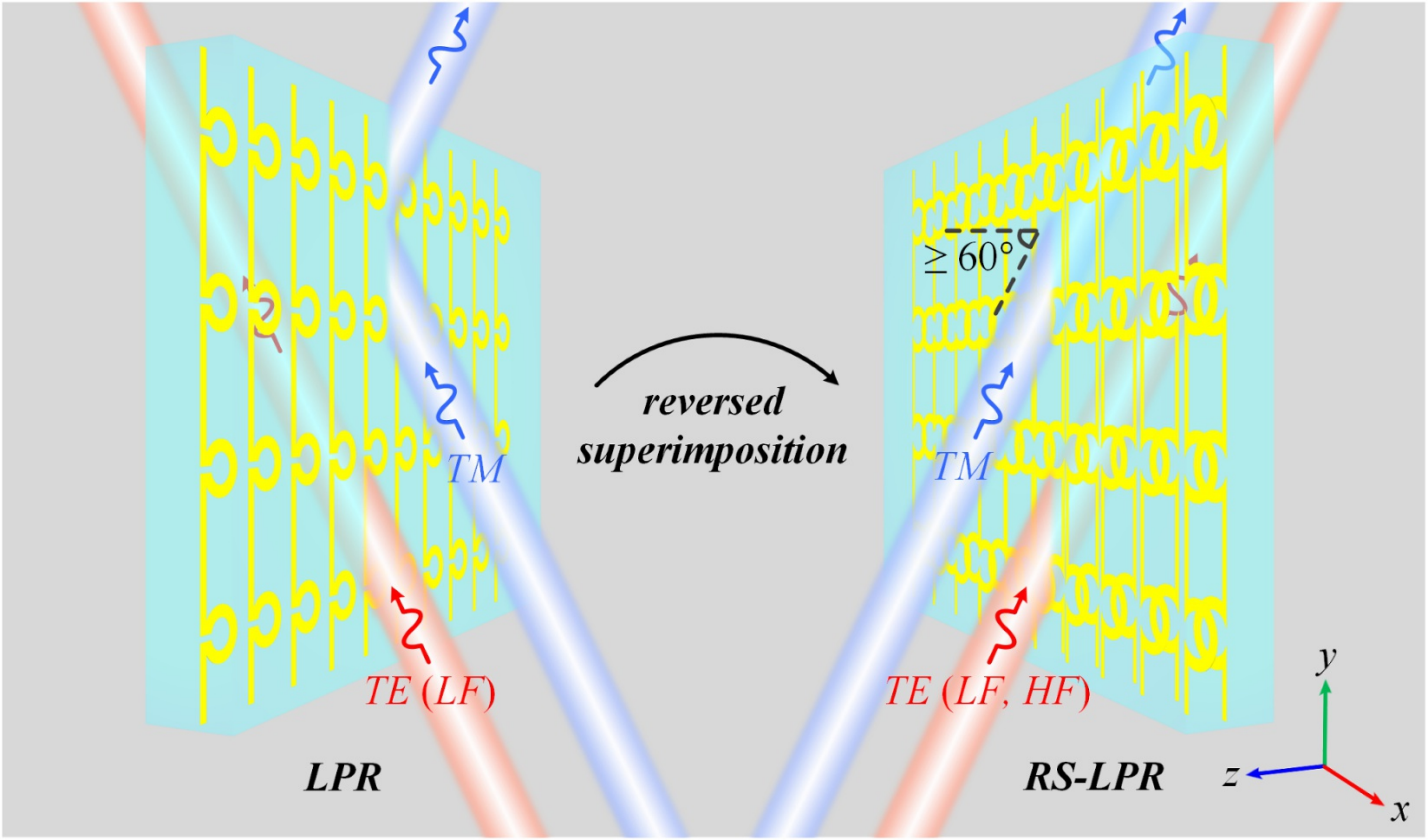
The LPR metasurface (the left) and the RS‐LPR metasurface (the right).

To solve the problem, we propose an idea of selective dispersion suppression (SDS) in this manuscript. It is to suppress the metasurface's undesired dispersion to widen its working range, while preserving the desired one to preserve the function. It is worth noting that in broadband achromatic metadevices, there is a similar design concept to widen the effective bandwidth, which is the integrated‐resonant unit element (IRUE) [30, 31, 32, 35]. Interestingly, the SDS here is exactly the opposite of the IRUE. The former is to suppress the resonance, while the latter is to promote the resonances.

As shown in Figure 1, through a method of reversed superimposition (RS), we have implemented the idea on the above LPR metasurface, and called the reversely superimposed metasurface as the RS‐LPR metasurface (the right in Figure 1). In the RS‐LPR metasurface, the undesired dispersions are well suppressed at both TE and TM polarizations, while the desired dispersion is preserved. It opens up the metasurface's TM‐polarized performance and widens its EB in the HF band, thus enabling the RS‐LPR metasurface to open the dual‐polarized and dual‐broadband EM window at large angles. We have well verified the SDS in the RS‐LPR metasurface through the spectral lines, the extracted EM parameters, and the monitored field. Moreover, we have compared the RS‐LPR metasurface with the original LPR metasurface and other related works, where the results show its superiority of dual‐polarization and broadband. The prototypes have been fabricated and measured, and the measured results are basically consistent with the simulated ones.

## 2 Analysis and Method

LPR metasurface: The unit of the LPR metasurface is illustrated in Figure 2a, which is an LMS along the *y*‐axis connected to a PR on the *xOy* plane. Here, the LMS and PR are both made of copper, and with a negligible thickness (t = 0.018 mm). When applied, the LPR metasurface is loaded in the substrate's middle layer. To meet applications, the ceramic matric composites (CMCs) function as the substrate's matric materials, which hold the relative permittivity and permeability of $\varepsilon_{1x}$ = $\varepsilon_{1y}$ = $\varepsilon_{1z}$ = 3.1 and $\mu_{1x}$ = $\mu_{1y}$ = $\mu_{1z}$ = 1.0 [36, 37, 38]. Here, $\varepsilon_{1i}$ and $\mu_{1i}$ are the relative permittivity and permeability along the *i*‐axis, respectively. At large angles, the CMC substrate's TM‐polarized transmission is high with the Brewster angle. However, due to the impedance mismatch, its TE‐polarized transmission is low [28].

**FIGURE 2:**
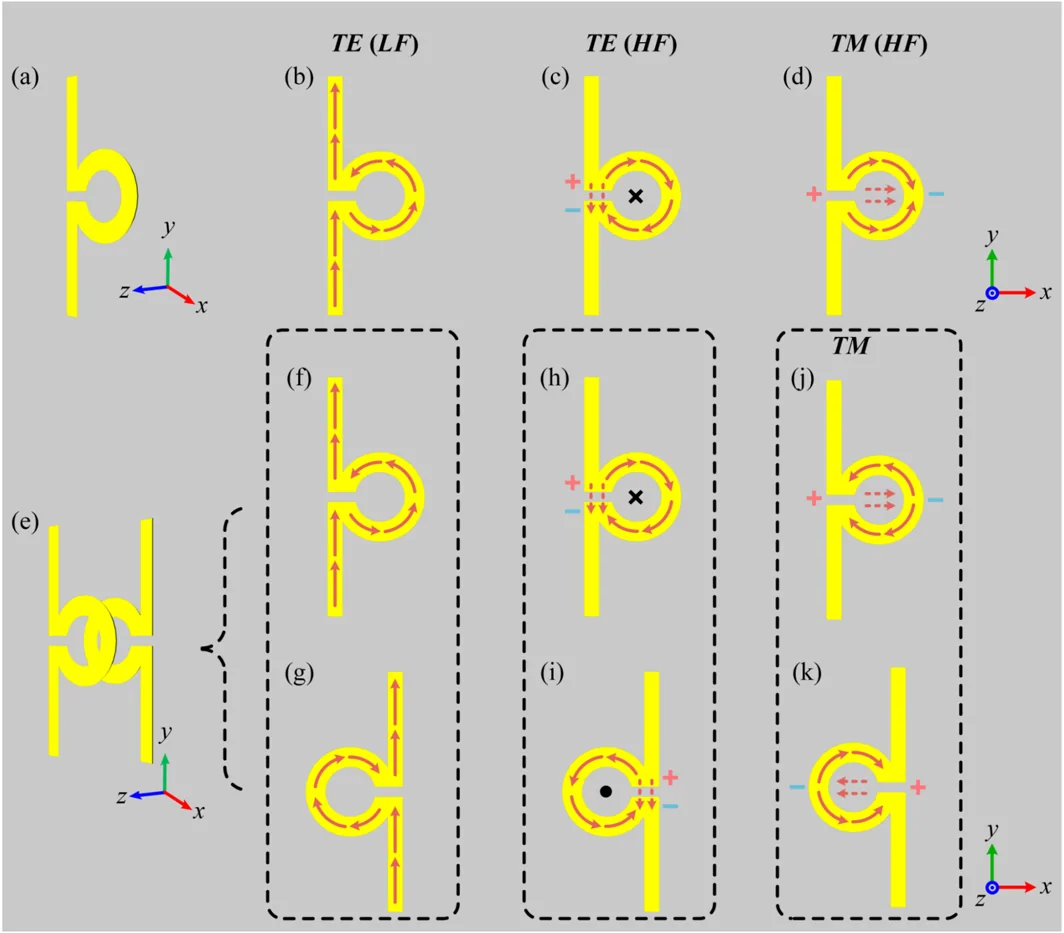
The illustration of (a) the LPR metasurface unit, (b) its plasma‐like oscillation, and its capacitive resonances responses along (c) the *x*‐axis and (d) *y*‐axis, respectively. The illustration of (e) the RS‐LPR metasurface unit, ((f) and (g)) its plasma‐like oscillation, and its capacitive resonances responses along ((h) and (i)) the *x*‐axis and ((j) and (k)) *y*‐axis in the upper and lower LPR units, respectively. Here, the red solid arrows are the induced currents, the red dotted arrows are the induced electrical field, and the black solid arrows are the induced magnetic field.

As introduced above, the LPR metasurface is to open the dual‐band EM window on the CMC substrate at large incident angles. In this manuscript, the EM wave is incident along the *z*‐axis's negative direction on the substrate. The TE polarization's electric field is always along the *y*‐axis for any incident angle, so is the TM polarization's magnetic field. In such a case, the substrate's TE‐polarized transmission is determined by its $\varepsilon_{1y}$, $\mu_{1x}$, and $\mu_{1z}$, while the TM‐polarized one is determined by the $\varepsilon_{1x}$, $\varepsilon_{1z}$, and $\mu_{1y}$. With the EM response, the LPR metasurface could disperse the $\varepsilon_{1y}$, $\varepsilon_{1x}$, and $\mu_{1z}$ [39, 40, 41]. However, due to the geometrical characteristics, it cannot disperse the $\varepsilon_{1z}$, $\mu_{1y}$, and $\mu_{1x}$.

Desired dispersion: At TE polarization, the currents along the *y*‐axis are induced by the incident electrical field in the LMSs. Meanwhile, in the low‐frequency (LF) working band, the PR has none of EM responses with its small electrical dimension, and there is nearly no current induced in it. Thus, the induced currents (red arrows) in LMSs go through the PRs, and thus flow continuously as shown in Figure 2b. The continuous currents generate the LMSs' plasma‐like oscillation, and induce the electrical field along the *y*‐axis to disperses the CMC substrate's $\varepsilon_{1y}$. It is the metasurface's desired dispersion in the LF band, which is to open the LF window ($W_{L}^{LPR}$) [28, 42]. In the HF working band, the LMSs' plasma‐like oscillation has attenuated, but the PR generates a capacitive resonance along the *y*‐axis. With the gap is only in one side of the PR, different electromotive forces are induced in the PR's left and right sides with TE‐polarization incident. Therefore, a circuit current is induced as shown in Figure 2c. With such a current, the PR works as a capacitor with the gap's two sides as the positive and negative electrodes, respectively. Thus, similar to that in the LF band, electrical field along the *y*‐axis is induced in the gap (dotted red arrows) to disperse the substrate's $\varepsilon_{1y}$. It supports the desired dispersion in the HF band, thereby opening the HF window ($W_{H}^{LPR}$).

Undesired dispersion: As shown in Figure 2c, in addition to the electrical field, the circuit current also induces a magnetic field (the black mark) in the PR's center along the *z*‐axis, and then disperses the substrate's $\mu_{1z}$. With $W_{H}^{LPR}$ only through the above desired dispersion of $\varepsilon_{1y}$, the dispersion of $\mu_{1z}$ here is undesired and reduces the EB of $W_{H}^{LPR}$. At TM polarization, where the incident electric field is in the *xOz* plane, the LMSs possess no response in both LF and HF bands due to their strong anisotropy. However, the PR generates a capacitive resonance along the *x*‐axis in the HF band. As shown in Figure 2d, with the TM‐polarized electrical field's component along the *x*‐axis, currents in the same direction are induced on the PR's upper and lower sides. Similar to that at TE polarization, the PR works as a capacitor with such homodromous current, where its left and right sides function as the positive and negative electrodes, respectively. Thus, electrical field along the *x*‐axis is induced within the PR, thereby dispersing the substrate's $\varepsilon_{1x}$. However, different from that at TE polarization, the homodromous currents here do not induce any magnetic field, so there is no dispersion of $\mu_{1z}$. As discussed above, the CMC substrate's TM‐polarized transmission is originally high at large angles. Thus, the dispersion of $\varepsilon_{1x}$ destroys the high transmission in the HF band as an undesired dispersion, and then makes $W_{H}^{LPR}$ ineffective at TM polarization.

To open up the LPR metasurface's TM‐polarized performance and widen its EB in the HF band, we are to implement the idea of SDS on it. According to the abovementioned facts, the metasurface disperses the medium through the induced field. Therefore, the induced field is preserved, the dispersion is preserved. Conversely, the induced field is suppressed, and the dispersion is suppressed. Thus, we propose to superimpose two LPR metasurfaces in a reverse direction along the *z*‐axis as shown in Figure 1, where the reversely superimposed metasurfaces are called the RS‐LPR metasurface. The unit of the RS‐LPR metasurface is illustrated in Figure 2e, where the PRs of the upper and lower LPRs are reversed with respect to the *y*‐axis, with their centers coinciding along the *z*‐axis.

Preserved desired dispersions: At TE polarization, the induced currents depend on the LMSs' plasma‐like oscillation in the LF band, and are none business of the PR with no EM response. Therefore, in the RS‐LPR unit, although the upper and lower PRs are reversed, the currents in the two LPRs are in the same direction as shown in Figure 2f,g, so is the electrical field along the *y*‐axis induced by them. Therefore, the total induced electrical field of the plasma‐like oscillation is preserved in the LF band, so is the desired dispersion of $\varepsilon_{1y}$. In the HF band, the induced currents are due to the PR's capacitive resonance. Therefore, with the two PRs reversed, the induced currents in them are also reversed (clockwise and counterclockwise) as shown in Figure 2h,i. However, even with the reversed currents, the induced electrical field in the two PRs' gaps are in the same direction. Similar to that in the LF band, it preserves the total induced electrical field along the *y*‐axis, and then, the desired dispersion of $\varepsilon_{1y}$ in the HF band.

Suppressed undesired dispersions: Different from the electrical field, the induced magnetic fields in the upper and lower PRs' centers are reversed (along the *z*‐axis's positive and negative directions) with the reversed circuit currents. It suppresses the total induced magnetic field, and thus the undesired dispersion of $\mu_{1z}$. At TM polarization, similar to that at TE polarization, the induced currents are due to the PR's capacitive resonance in the HF band. Therefore, the induced currents in the upper and lower PRs are also reversed as shown in Figure 2j,k. However, different from that at TE‐polarization, with the reversed currents, the induced electrical field along the *x*‐axis within two PRs are reversed here. Thus, the total induced electrical field is suppressed, so is the undesired dispersion of $\varepsilon_{1x}$.

Importantly, the SDS is not only applicable to the LPR metasurface but also to almost all metasurfaces, especially those with asymmetric architectures such as split‐ring resonators (SSRs). Moreover, according to the metasurfaces' mechanisms, the RS design above is the general method to implement the SDS. The superimposed metasurfaces should be aligned in the same direction along their asymmetric axis and in the opposite directions along the symmetric axis.

## 3 Verification

To verify the SDS in the RS‐LPR metasurface, we have designed the LPR and RS‐LPR metasurfaces for a 5‐mm‐thick CMC substrate, respectively. The units of the LPR‐loading and RS‐LPR‐loading substrates are illustrated in Figure 3a–f, respectively. Meanwhile, the values of their structural parameters are given in Tables 1 and 2, respectively, where t represents the thickness of the LPR. Additionally, because the upper and lower LPRs in the RS‐LPR are the same, only the upper one is shown in Figure 3e.

**FIGURE 3:**
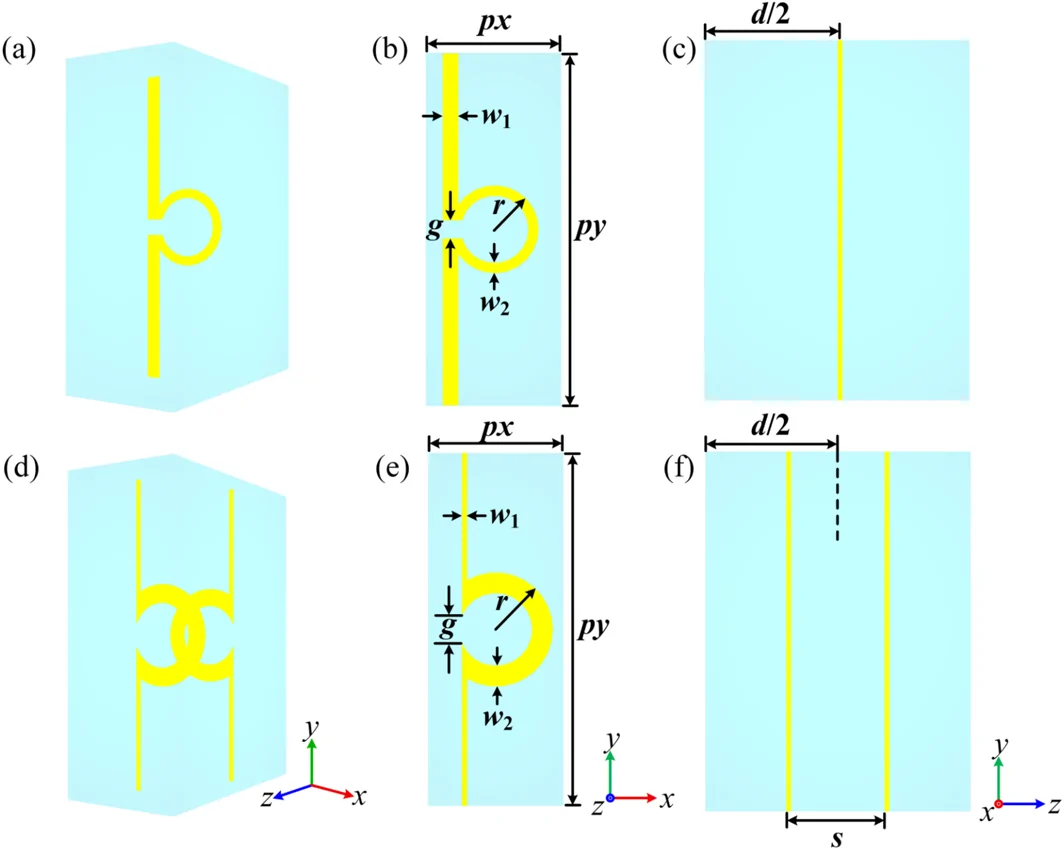
The three views of the (a–c) LPR‐loading and (d–f) RS‐LPR‐loading substrates' units.

**TABLE 1:** The values of the structural parameters of the LPR metasurface.

*px*	*py*	*w*_1_	*w*_2_	*r*	*g*	*t*	*d*
1.8	5.5	0.3	0.2	0.55	0.3	0.018	5.0

**TABLE 2:** The values of the structural parameters of the RS‐LPR metasurface.

*px*	*py*	*w*_1_	*w*_2_	*r*	*g*	*t*	*d*	*s*
2.6	6.8	0.1	0.4	1.1	0.5	0.018	5.0	2

Transmission spectral lines and extracted parameters: As shown in Figure 4a,b, we have simulated the TE‐ and TM‐polarized transmission spectral lines of the empty CMC substrate, the LPR‐loading, and the RS‐LPR‐loading substrates at the incident angle of $\theta_{i}$ = 80°, respectively. Meanwhile, as shown in Figure 4c,d, we have extracted the $\varepsilon_{1y}$, $\varepsilon_{1x}$, and $\mu_{1z}$ of the LPR‐ and the RS‐LPR‐loading substrate, which is through the method in Reference [43]. Here, Ku and Ka bands are the LF and HF bands to meet applications, respectively. Moreover, the results extracted through the algorithm in Reference [44] may have some inaccuracies, because the substrate loaded with the metasurface is not the isotropic medium in the strict sense. However, according to Snell's law, the spectral trend of the extracted parameters' real parts could still well prove the design's working mechanism.

**FIGURE 4:**
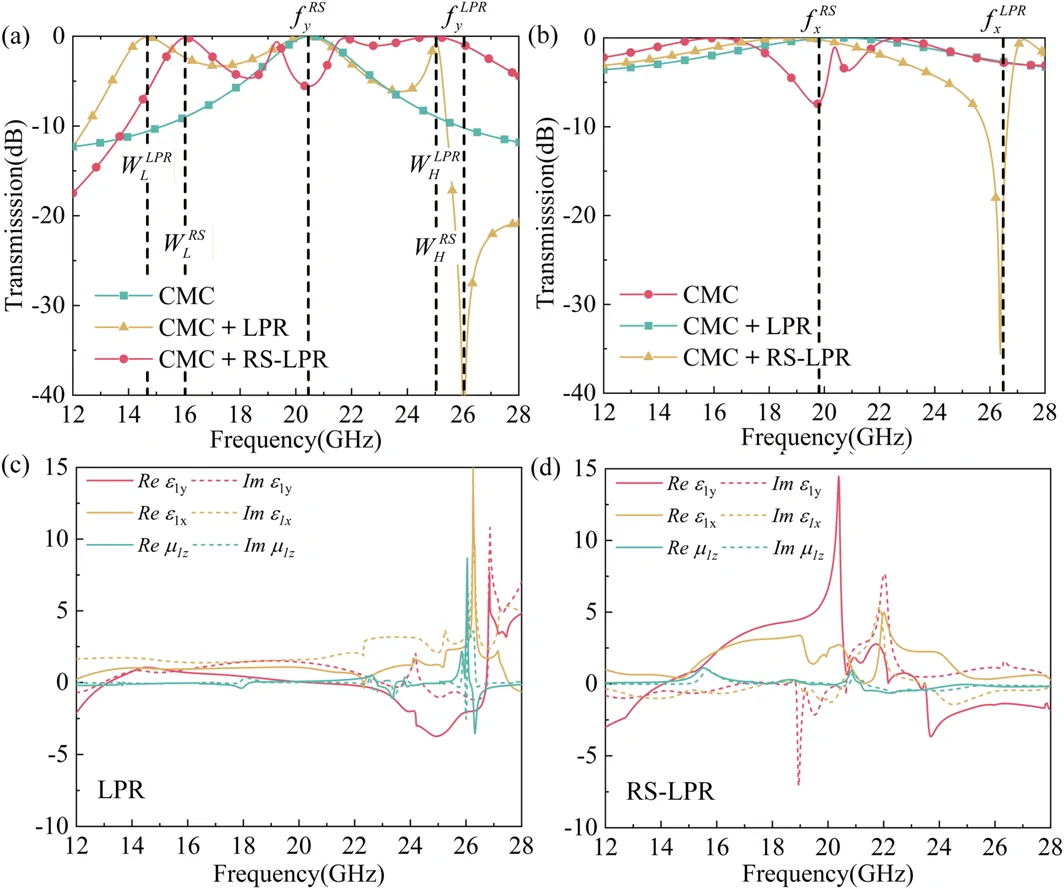
The simulated (a) TE‐ and (b) TM‐polarized transmission spectral lines of the empty CMC substrate. The LPR‐, and RS‐LPR‐loading substrates at the incident angle of $\theta_{i}$ = 80°. The extracted $\varepsilon_{1y}$, $\varepsilon_{1x}$, and $\mu_{1z}$ of the (c) LPR‐ and (d) RS‐LPR‐loading substrates, where the solid and dotted lines represent the real and imaginary parts, respectively.

At TE polarization, as shown in Figure 4a, the empty substrate's transmission (cyan line with square marks) is high at around 20.4 GHz. According to the calculation, it is the substrate's 1st‐order half‐wave wall [45, 46]. Meanwhile, in most of the LF and HF bands, its transmission is very low due to the impedance mismatch. After loading the LPR metasurface (yellow line with triangle marks), $W_{L}^{LPR}$ (14.7 GHz) and $W_{H}^{LPR}$ (25.0 GHz) are opened on the substrate through the desired dispersion of $\varepsilon_{1y}$. Moreover, as shown in Figure 4c, with the plasma‐like oscillation being a nonresonant response, the dispersion curve of $\varepsilon_{1y}$ is gentle in the LF band, and it endows $W_{L}^{LPR}$ with a wide EB [42]. With the window's effective transmission as ≥ 3 dB here, the EB of $W_{L}^{LPR}$ is 13.8–16.6 GHz at $\theta_{i}$ = 80°, and its fractional bandwidth (FBW) is 18.4%. Here, the FBW is expressed as $f_{h}-f_{l}/f_{c}$, where $f_{l}$, $f_{h}$, and $f_{c}$ represent the lower, higher, and center frequencies of EB, respectively. However, different from the plasma‐like oscillation, the PR's capacitive resonance is resonant, and the dispersions of $\varepsilon_{1y}$ and $\mu_{1z}$ have both vibrated at $f_{y}^{LPR}$ = 26.0 GHz, where $f_{y}^{LPR}$ is the LPR's capacitive resonance frequency along the *y*‐axis. Therefore, a transmission dip appears at $f_{y}^{LPR}$ as shown Figure 4a, which is with a high‐quality (Q) factor and a transmission ≤ 40 dB. Importantly, with the vibration, the dispersion curves of $\varepsilon_{1y}$ and $\mu_{1z}$ are steep near $f_{y}^{LPR}$, which results in the EB of $W_{H}^{LPR}$ being very narrow. Here, the EB of $W_{H}^{LPR}$ is 24.8–25.2 GHz, and its FBW is only 1.6%, which is much smaller than that of $W_{L}^{LPR}$. As shown in Figure 4d, for the RS‐LPR loading substrate, the desired dispersion of $\varepsilon_{1y}$ is preserved in both LF and HF bands with $f_{y}^{RS}$ = 20.4 GHz, where $f_{y}^{RS}$ is RS‐LPR's capacitive resonance frequency along the *y*‐axis. Thus, as shown in Figure 4a, the LF and HF windows are also opened (red line with circular marks), which are at 16.0 GHz ($W_{L}^{RS}$) and 25.0 GHz ($W_{H}^{RS}$), respectively. Moreover, similar to that in the LPR‐loading substrate, $W_{L}^{RS}$ has a wide EB through the gentle dispersion of $\varepsilon_{1y}$ by the plasma‐like oscillation, and a transmission dip appears at $f_{y}^{RS}$ due to $\varepsilon_{1y}$'s dispersion vibration. However, different from that in the LPR‐loading substrate, the dispersion of $\mu_{1z}$ has been well suppressed in the RS‐LPR loading substrate, which has shown no obvious vibration in the spectrum. Thus, the dip at $f_{y}^{RS}$ has a higher transmission (≥ 9 dB) and a lower Q factor than that at $f_{y}^{LPR}$. Importantly, with no obvious vibration, the dispersion curve of $\mu_{1z}$ is gentle in the whole spectrum. Therefore, the EB of $W_{H}^{RS}$ (22.8–26.7 GHz) is much wider than that of $W_{H}^{LPR}$, and its FBW has reached 15.1%, which is nearly 943.8% of that of $W_{H}^{LPR}$.

At TM polarization, as shown in Figure 4b, the transmission of the empty CMC substrate is high in the whole spectrum with the Brewster angle. However, after loading the LPR metasurface, a dip appears at 26.3 GHz, which is also with a high Q factor and a low transmission (≤ 40 dB). With the dip, the substrate's originally high transmission is destroyed in the HF band, thus leading to $W_{H}^{LPR}$ being ineffective at TM polarization. As shown in Figure 4c, similar to that at TE polarization, it is due to the dispersion vibration of $\varepsilon_{1x}$ at $f_{x}^{LPR}$ = 26.3 GHz, where $f_{x}^{LPR}$ is the LPR's capacitive resonance frequency along the *x*‐axis. For the RS metasurface‐loading substrate, the dip also appears at $f_{x}^{RS}$ = 19.7 GHz, where $f_{x}^{RS}$ is the RS‐LPR's capacitive resonance frequency along the *x*‐axis. However, as shown in Figure 4d, the dispersion of $\varepsilon_{1x}$ is suppressed in the RS‐LPR‐loading substrate compared with that in the LPR‐loading one, and its vibration is much slighter than that at $f_{x}^{LPR}$. Therefore, as shown in Figure 4b, the dip is with a higher transmission (≥ 7.5 dB) and lower Q factor than that at $f_{x}^{LPR}$. With such a slight dip, the substrate's high TM‐polarized is preserved in the HF band. Thus, different from $W_{H}^{LPR}$, $W_{H}^{RS}$ is also effective at TM polarization. By the way, compared with the $W_{L}^{LPR}$, $f_{y}^{LPR}$, and $f_{x}^{LPR}$ of the LPR‐loading substrate, $W_{L}^{RS}$, $f_{y}^{RS}$, and $f_{x}^{RS}$ of the RS‐LPR loading one are at higher frequencies. It is because the metasurface's plasma‐like frequency and the total capacitance and inductance are both increased with the additional layer of LPRs [24, 39].

Monitored field: To further verify the SDS in the RS‐LPR metasurface, we have monitored the surface currents in its upper and lower LPRs at $W_{L}^{RS}$, $f_{y}^{RS}$, and $f_{x}^{RS}$ at $\theta_{i}$ = 80°. As shown in Figure 5a,b, in both upper and lower LPRs, the currents in the LMS go through the PR and then flow continuously at $W_{L}^{RS}$ (See the black dotted arrows.). Moreover, the currents in the two LPRs are in the same direction. At $f_{y}^{RS}$, different from that at $W_{L}^{RS}$, the currents in the PR may be opposite to that in the LMS as shown in Figure 5e, which is due to the PR's capacitive resonance. Importantly, as shown in Figure 5d,e, the currents in the lower and upper PRs are reversed (clockwise and counterclockwise, respectively). As shown in Figure 5f,g, homodromous currents are induced in the PR's two sides at $f_{x}^{RS}$. Additionally, similar to that at $f_{y}^{RS}$, the currents in the two PRs are reversed. In summary, the above monitored currents are basically consistent with our analysis (see Figure 2f–k), and could verify the SDS in the RS‐LPR. By the way, at all monitored frequencies, the currents in the LMS are more obvious than that in the PR, which is due to the LMS's narrow width ($w_{1}$)

**FIGURE 5:**
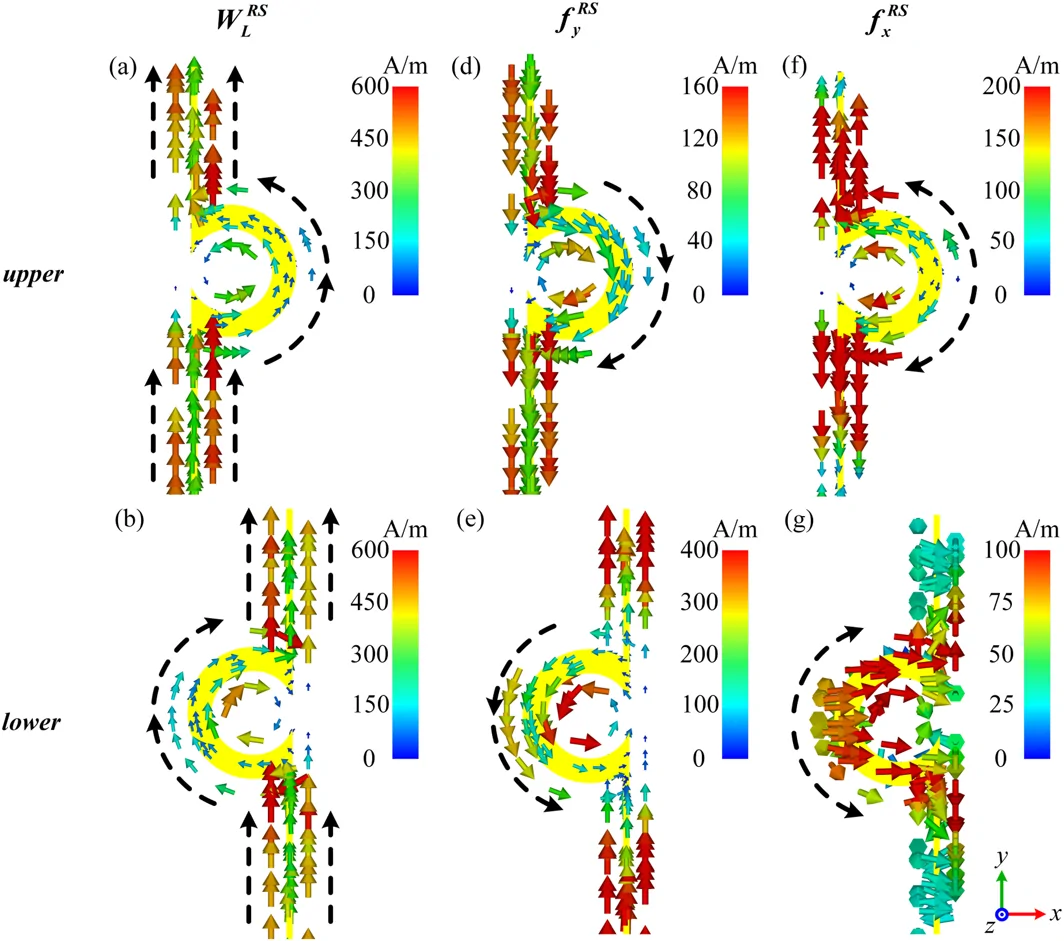
The monitored surface currents in the RS‐LPR metasurface's upper and lower LPRs at ((a) and (b)) $W_{L}^{RS}$, ((c) and (d)) $f_{y}^{RS}$, and ((e) and (f)) $f_{x}^{RS}$ at $\theta_{i}$ = 80°, respectively, where the currents' direction is indicated by the black dotted arrows.

In addition to the currents, we have also monitored the electrical field at above frequencies. As shown in Figure 6a,b, electrical field along the *y*‐axis is induced near the LMS at $W_{L}^{RS}$, and the upper and lower induced electrical field is in the same direction. At $f_{y}^{RS}$, as shown in Figure 6c,d, electrical field along the *y*‐axis is induced in the PRs' gaps. Additionally, the induced electrical field in the two gaps are in the same direction (See the black dotted square box.). As shown in Figure 6e,f, the electrical field along the *x*‐axis is induced within the two PRs at $f_{x}^{RS}$. However, different from that at $f_{y}^{RS}$, the upper and lower induced electrical fields are reversed here. Moreover, we have monitored the magnetic field in the RS‐LPR unit at $f_{y}^{RS}$ and $f_{x}^{RS}$, respectively. As shown in Figure 6g, magnetic field along the *z*‐axis is obviously induced in both the upper and lower PRs' centers. Importantly, the upper and lower induced magnetic fields are reversed. Meanwhile, different from that at $f_{y}^{RS}$, nearly no magnetic field along the *z*‐axis is induced in the PRs at $f_{x}^{RS}$, as shown in Figure 6h. Thus, in addition to the monitored currents, the monitored electrical and magnetic fields are also consistent with our analysis (see Figure 2f–k), and could verify the SDS in the RS‐LPR metasurface.

**FIGURE 6:**
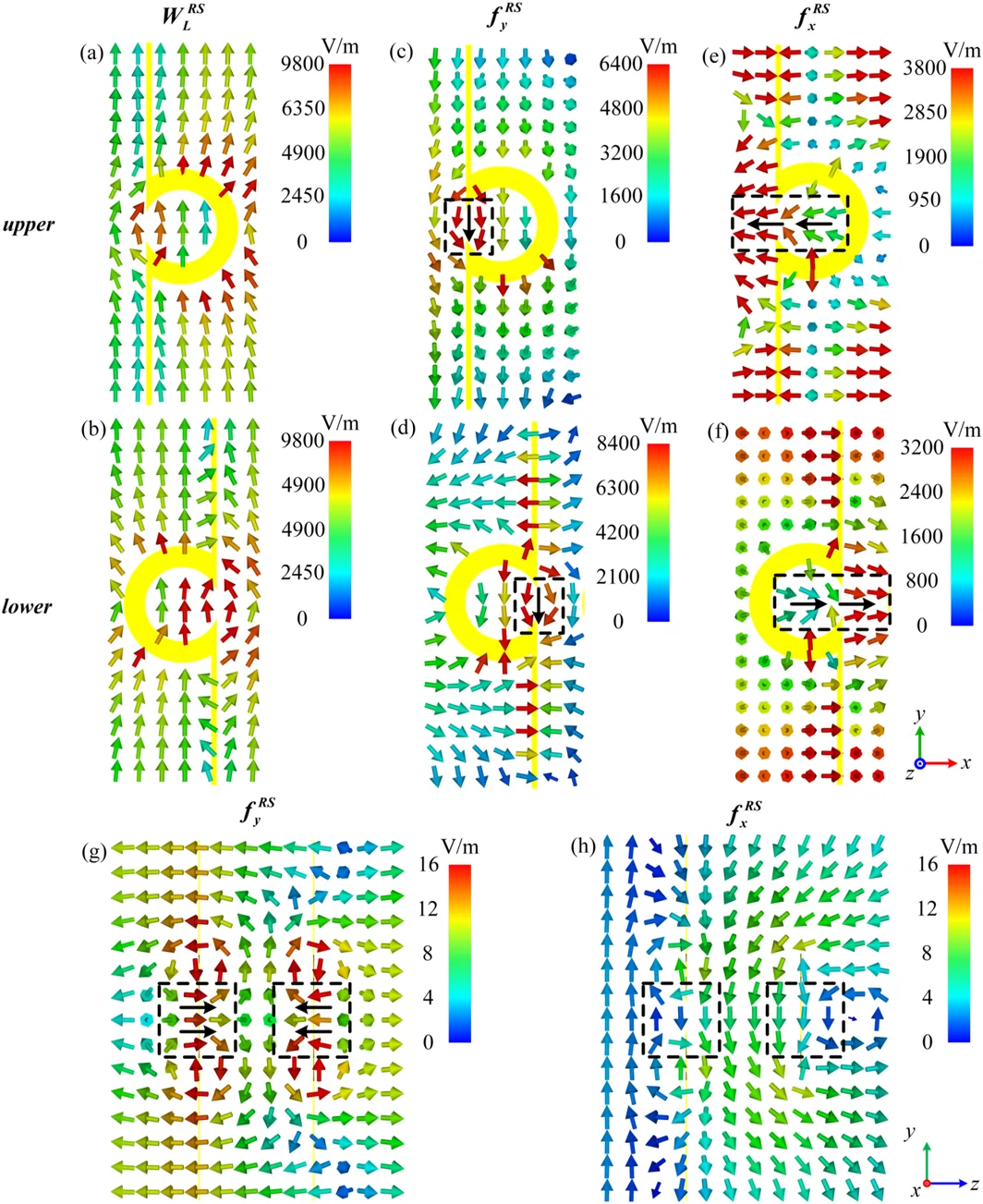
The monitored electrical field in the RS‐LPR metasurface's upper and lower LPRs at ((a) and (b)) $W_{L}^{RS}$, ((c) and (d)) $f_{y}^{RS}$, and ((e) and (f) $f_{x}^{RS}$ at $\theta_{i}$ = 80°, respectively. The monitored magnetic field in the RS‐LPR unit at (g) $f_{y}^{RS}$ and (h) $f_{x}^{RS}$ at $\theta_{i}$ = 80°, respectively. Here, the induced field's direction is indicated by the black solid arrows.

## 4 Performance

Simulation: In order to fully show the RS‐LPR metasurface's performance, we have simulated the TE‐ and TM‐polarized transmission spectrum of the empty CMC plate and the LPR‐loading and the RS‐LPR loading substrates within $\theta_{i}\epsilon$ [60°∼85°], respectively. As shown in Figure 7a–f, both the LPR and RS‐LPR metasurfaces could open the LF and HF windows on the CMC substrate. However, as shown in Figure 7c,e, $W_{H}^{RS}$ is obviously with a wider EB than $W_{H}^{LPR}$ in the large‐angle spectrum. Additionally, as shown in Figure 7d,f, compared with $W_{H}^{LPR}$, $W_{H}^{RS}$ is effective at TM polarizaition with the substrate's high transmission preserved.

**FIGURE 7:**
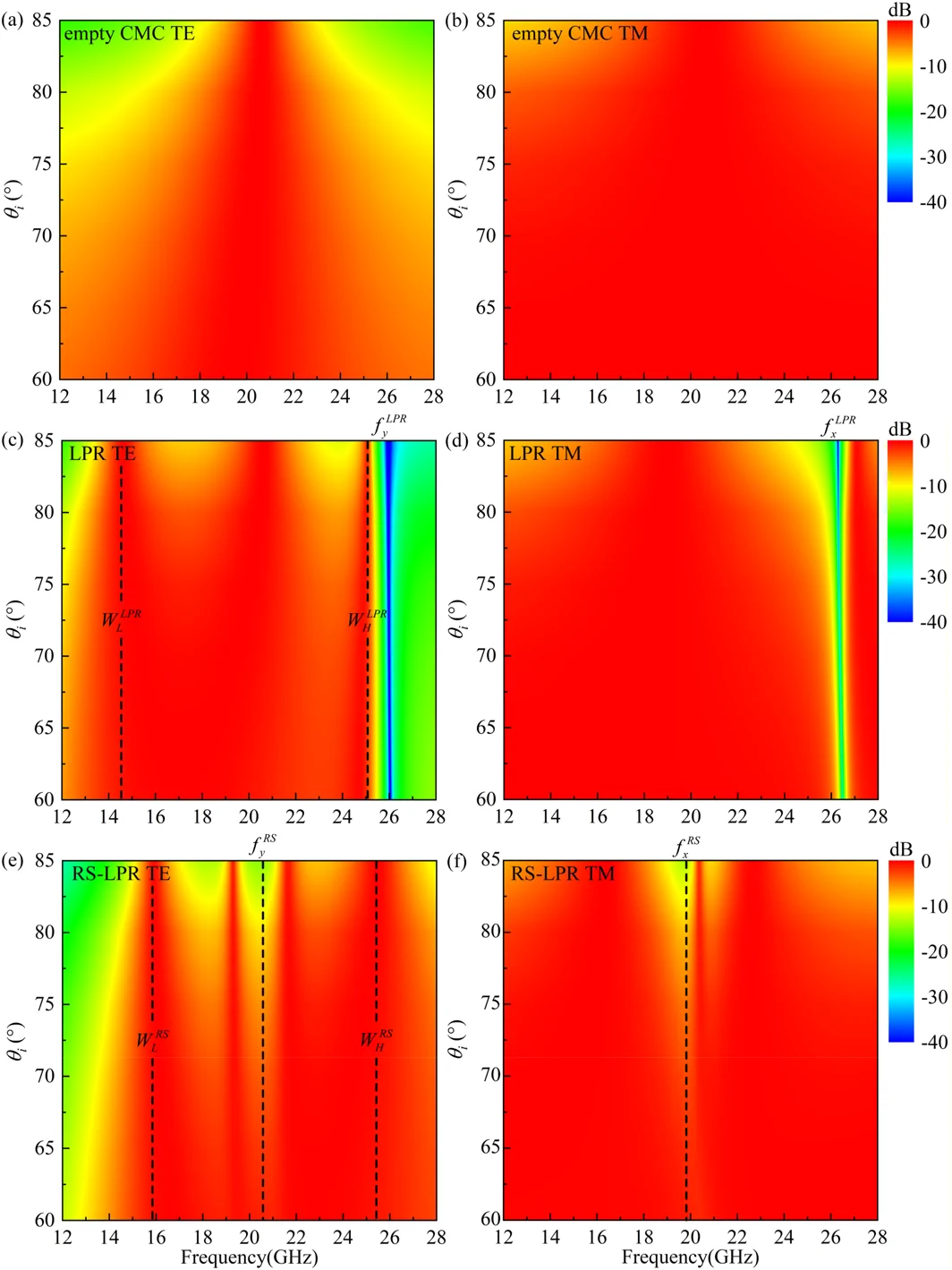
The simulated TE‐ and TM‐polarized transmission spectrum of the ((a) and (b)) empty CMC substrate and ((c) and (d)) LPR‐ and ((e) and (f)) RS‐LPR‐metasurface‐loading substrates within $\theta_{i}\epsilon$ [60°∼85°], respectively.

Due to the limited precision of current technologies, errors usually occur in metasurface processing. Thus, the robustness of the metasurface's performance of these errors will directly determine the design's practical application value. Therefore, based on the current technologies, several parameters prone to errors and their corresponding error ranges are summarized. Further, the effect of these errors on the RS‐LPR metasurface's performance has been analyzed through series of simulations, where the results prove the great robustness of the RS‐LPR metasurface. The detailed results are given in Supporting Information S1.

In order to further demonstrate the superiority of the RS‐LPR metasurface, it is compared with the LPR metasurface and several other works reported in the last 5 years that could also open the EM window at large angles [16, 17, 18, 19, 20]. With the effective transmission defined as ≥ 3 dB, every work's effective polarization type, EBs, and FBWs at $\theta_{i}$ = 80° are shown in Table 3. Moreover, for a better comparison, the works' design architectures (DAs) are also given. Obviously, compared with other works, the RS‐LPR metasurface not only could open the dual‐band window for dual polarization, but also possess two wide FBWs.

**TABLE 3:** Comparison between the RS‐LPR metasurface and other works.

	Polarization	EB	FBW	DA
RS‐LPR	TE and TM	22.8–26.7 GHz	13.6%	Dual‐layer LPRs
		28.72–29.58 GHz	15.1%	
LPR	TE	13.8–16.6 GHz	18.4%	Single‐layer LPRs
		24.8–25.2 GHz	1.6%	
Reference [16]	TE and TM	5.68–6.43 GHz	12.4%	Grid and X‐shaped resonators
Reference [17]	TE and TM	33.01–35.29 GHz	6.7%	Dual‐layer grid
Reference [18]	TE and TM	277.12–292.42 GHz	5.4%	Grid and circular resonators
Reference [19]	TE and TM	76.31–78.94 GHz	3.4%	Grid and cruciform resonators
Reference [20]	TE	6.85–7.03 GHz	2.6%	Grid and rectangular resonators

Measurement: To further verify the performance of the RS‐LPR metasurface, the prototypes were designed, processed, and measured. For the LPR‐loading substrate, we have first processed a 2.5‐mm‐thick CMC plate, and etched the LPR metasurface on one side of it through the printed circuit board technology. Then, we have stuck the above plate with the other empty plate of the same thickness, with the metasurface in the sandwich layer. The LPR‐metasurface‐etching plate and the local enlarged image of its surface are shown in Figure 8a,b, respectively. For the RS‐LPR‐loading substrate, we have processed a 2.0‐mm‐thick CMC plate, and then etched the upper and lower LPR metasurfaces on its both sides. Then, we have sandwiched it in the middle with two 1.5‐mm‐thick CMC plates. The RS‐LPR‐metasurface‐etching plate and the local enlarged image of its surface are shown in Figure 8c,d, respectively. For the above prototypes, the adjacent CMC plates were glued together with the glass cement. In addition, a 5.0‐mm thick empty CMC plate is processed as a comparison prototype. To match the measured equipment and angles, all prototypes are processed as 300 mm × 500 mm.

**FIGURE 8:**
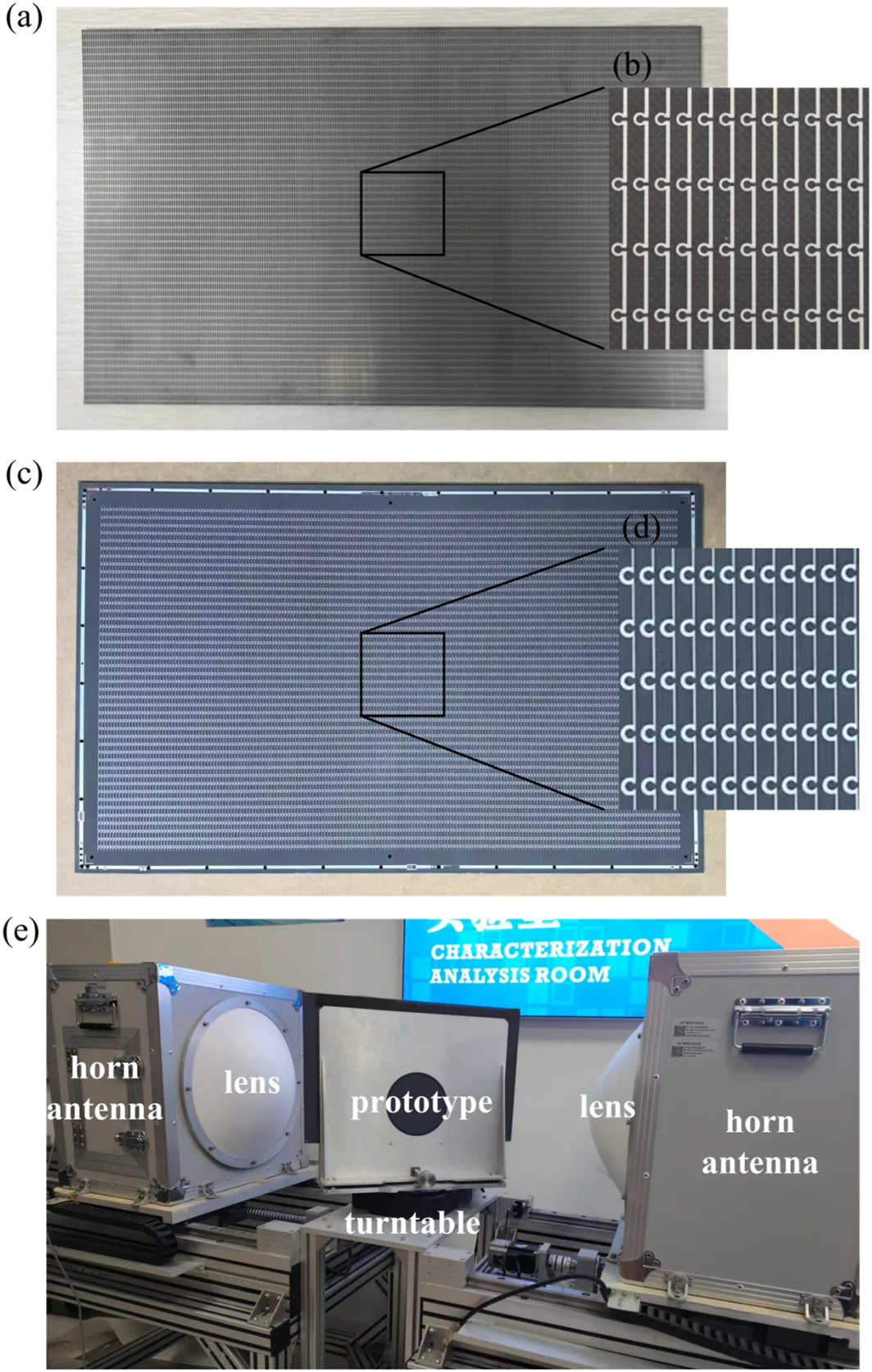
(a) The processed CMC plate with the LPR metasurface etched on one of its surfaces and (b) the locally enlarged image of its surface. (c) The processed CMC plate with the RS‐LPR metasurface etched on one of its surfaces and (d) the locally enlarged image of its surface. (e) The measured environment.

As shown in Figure 8e, the above prototypes were measured in the lens antenna measurement system, respectively. Here, the horn antennas were used as the transmitting and receiving ports, and the prototype was positioned on the turntable between the two lenses. The measured results of the three prototypes are shown in Figure 9a–f, which are basically consistent with the simulation ones in Figure 7a–f. For the error between the measured and simulated results, it may be due to the clutter and losses in transmission lines.

**FIGURE 9:**
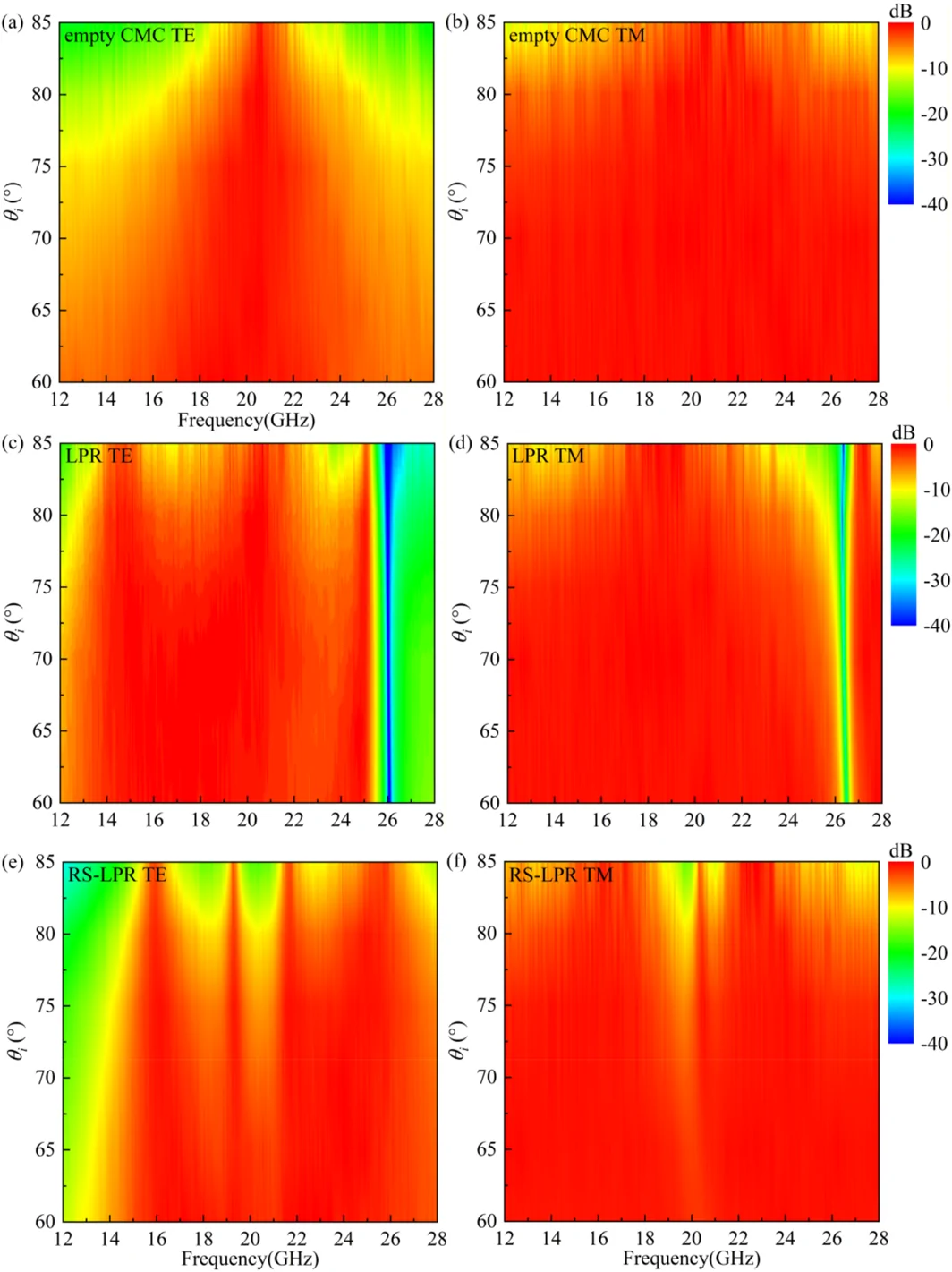
The measured TE‐ and TM‐polarized transmission spectrum of the ((a) and (b)) empty CMC substrate, and ((c) and (d)) LPR‐ and ((e) and (f)) the RS‐LPR‐metasurface‐loading substrates within $\theta_{i}\epsilon$ [60°∼85°], respectively.

## 5 Conclusion

In this manuscript, we have proposed to suppress the metasurface's undesired dispersion to widen its working range, while preserving the desired one to preserve the EM function, and called the idea SDS. Through the method of RS, we have implemented the idea on the LPR metasurface in Li et al. The LPR metasurface is for the dual‐band EM window at large angles, but could only work for TE polarization and has a narrow EB in the HF band. However, through the SDS, its TM‐polarized performance is opened up and the EB is widened in the HF band. Thus, the RS‐LPR metasurface could open the dual‐polarized and dual‐broadband EM window at large angles. Through the transmission spectral lines, extracted EM parameters, and monitored field, we have verified the SDS in the RS‐LPR metasurface. Moreover, we have compared the RS‐LPR metasurface with the original LPR metasurface and other related works, and the results have shown its superiority of dual polarization and broadband. The prototypes were fabricated and measured, where the measured results were basically consistent with the simulated ones. This work may provide new ideas for dual‐polarization and broadband metasurfaces, and find wide applications in 5 G/6G base stations, radar systems, and others.

## 6 Methods

Simulation Section: With the assistance of the commercial software CST microwave studio, the S_Zmax(1), Zmin(1)_ (transmission) of unit cells of proposed anti‐reflective metasurface and the substrate is simulated with frequency‐domain Solver and boundary conditions that X‐unit cell, Y‐unit cell and Z‐open add space. And the surface current and energy flow distributions are obtained by the field monitor.

## Author Contributions

**Tiefu Li:** software, methodology, conceptualization, data curation, investigation, validation, formal analysis, supervision, visualization, project administration, resources, writing – original draft, writing – review and editing. **Jinwei Chen:** software. **Cunqian Feng:** writing – review and editing. **Jian Liu:** writing – review and editing. **Yuxiang Jia:** writing – review and editing. **Chang Ding:** writing – review and editing. **Yajuan Han:** writing – review and editing. **Xinmin Fu:** writing – review and editing. **Jie Yang:** writing – review and editing. **Zhenxu Wang:** writing – review and editing. **Song Zhao:** software. **Zhaotang Liu:** writing – review and editing. **Zhihui Zhang:** writing – review and editing. **Jiafu Wang:** writing – review and editing, funding acquisition.

## Acknowledgements

This work was supported by the National Joint Fund for Regional Innovation and Development of Science and Technology (Grant U24A20224), the National Natural Science Foundation of China (Grants 52272101 62401615, 62401617, 62301596, and 62401616); the Shanxi Province Scientific and Technology Innovation Team Foundation of Shaanxi Province (Grant 2023‐JC‐QN‐0740).

## Conflicts of Interest

The authors declare no conflicts of interest.

## Supporting information


Supporting Information S1

## Data Availability

The data that support the findings of this study are available from the corresponding author upon reasonable request.
