# Supplementary material for: A Selective Dispersion–Suppressed Metasurface for Dual‐Polarization and Dual‐Broadband Electromagnetic Window at Large Angles
Source: Nanophotonics. 2026 Jul 20;15(14):e70166. doi: 10.1002/nap2.70166 (PMC13642185; doi:10.1002/nap2.70166)
Supplement: Supplementary file 1 [file NAP2-15-e70166-s001.docx]

Supplement Materials

**ROBUSTNESS ANALYSIS**

According to current processing technologies, it is not the units’ period ($px$ and $py$) but the structural parameters that are prone to errors in the metasurface processing. Therefore, the RS-LPR metasurface’s robustness of the errors of $m$ $r$, $w_{1}$ and $w_{2}$ is analyzed here, where $m$ is the misalignment of upper and lower LPRs’ misalignment along the $x$-axis.

Here, the error ranges of the above parameters are first summarized, which are $m\epsilon$[0.1, 0.3], $r\epsilon$[1.05, 1.15], $w_{1}\epsilon$[0.05, 0.15] and $w_{2}\epsilon$[0.35, 0.45] in millimeters. Then, as shown in Figure S1 and S2, the RS-LPR-metasurface-loading substrate’s TE- and TM-polarized transmission at $\theta_{i}$ = 80$^{\circ}$ within the error range of each parameter is simulated, respectively. Obviously, as the parameters vary within the error range, the transmission spectrum only undergoes a slight frequency shift without any significant change. Especially for $m$, the substrate’s transmission remains almost constant. In summary, the above simulation results prove the RS-LPR metasurface’s great robustness, and thus its application value.

Figure S1 The simulated TE-polarized transmission spectral lines of the RS-LPR-loading substrates at the incident angle of $\theta_{i}$ = 80$^{\circ}$ within (a) $m\epsilon$[0.1, 0.3], (b) $r\epsilon$[1.05, 1.15], (c) $w_{1}\epsilon$[0.05, 0.15] and (d) $w_{2}\epsilon$[0.35, 0.45].

Figure S2 The simulated TM-polarized transmission spectral lines of the RS-LPR-loading substrates at the incident angle of $\theta_{i}$ = 80$^{\circ}$ within (a) $m\epsilon$[0.1, 0.3], (b) $r\epsilon$[1.05, 1.15], (c) $w_{1}\epsilon$[0.05, 0.15] and (d) $w_{2}\epsilon$[0.35, 0.45].
